# Long term safety and tolerability of Tafluprost 0.0015% vs Timolol 0.1% preservative-free in ocular hypertensive and in primary open-angle glaucoma patients: a cross sectional study

**DOI:** 10.1186/s12886-017-0534-z

**Published:** 2017-08-03

**Authors:** Teresa Rolle, Roberta Spinetta, Raffaele Nuzzi

**Affiliations:** Eye Clinic, Department of Surgical Sciences, University of Torino, Via Juvarra 19, 10122 Torino, Italy

**Keywords:** Primary open angle glaucoma, Ocular surface, Timolol preservative free, Tafluprost, In vivo confocal microscopy

## Abstract

**Background:**

The effects of preservatives of antiglaucoma medications on corneal surface and tear function have been widely shown in literature; it’s not the same as regards the active compounds themselves. The purpose of our study was to compare Ocular Surface Disease (OSD) signs and symptoms of Tafluprost 0.0015% versus preservative free (PF) Timolol 0.1% eyedrops in ocular hypertensive (OH) and in primary open-angle glaucoma (POAG) patients.

**Methods:**

A cross-sectional study included patients in monotherapy for at least 36 months with Tafluprost 0.0015% (27) or PF Timolol 0.1% (24) and 20 healthy age and sex-matched volunteers. All subjects underwent clinical tests (Schirmer I and break-up time), in vivo confocal microscopy (IVCM) and were surveyed using Ocular Surface Disease Index (OSDI) and Glaucoma Symptoms Scale (GSS) questionnaires. The groups were compared with ANOVA, Kruskal-Wallis test, t-test, Mann-Whitney test and Bonferroni’s adjustment of *p*-values.

**Results:**

No significant differences were found in questionnaires scores, clinical tests, IVCM variables between therapy groups. Tafluprost 0.0015% group showed significantly higher OSDI score, basal epithelial cells density, stromal reflectivity, sub-basal nerves tortuosity (*p* = 0.0000, 0.037, 0.006, 0.0000) and less GSS score, number of sub-basal nerves (*p* = 0.0000, 0.037) than controls but similar clinical tests results (*p* > 0.05). PF Timolol group had significantly higher OSDI score, basal epithelial cells density, stromal reflectivity and sub-basal nerve tortuosity (*p* = 0.000, 0.014, 0.008, 0.002), less GSS score, BUT and number of sub-basal nerves (*p* = 0.0000, 0.026, 0.003) than controls.

**Conclusions:**

Compared to PF Timolol 0.1%, Tafluprost 0.0015% showed similar safety with regards to tear function and corneal status and a similar tolerability profile. Both therapy groups show some alterations in corneal microstructure but no side effects on tear function except for an increased tear instability in PF Timolol 0.1% group. Ophtalmologists should be aware that even PF formulations may lead to a mild ocular surface impairment.

## Background

Glaucoma is an optic neuropathy with a multifactorial etiology; the increased intraocular pressure (IOP) is the only risk factor on which we can act and is thought to play the main role in pathogenesis [1]. Medical treatment is usually the first therapeutic choice to treat POAG and at present prostaglandin analogs (PGA) and beta-blockers are the initial therapies of choice worldwide [2].

There is growing evidence that the long term use of antiglaucoma drugs may induce ocular surface toxicity [3, 4], causing ocular surface disease and ocular discomfort [5, 6] which may affect patient’s quality of life and compliance.

Previous studies have already pointed out that toxic and proinflammatory effects of antiglaucoma ophtalmic solutions are mainly due to preservatives [3, 4]; so to reduce discomfort and long term side effects and to increase compliance to the topical therapy, preservative free formulations have been developed as they have shown to have an equivalent efficacy to the preserved ones [7, 8].

PGA are a first line therapy option in treating elevated intraocular pressure because of their good efficacy and positive safety profile [9]. Tafluprost 0.0015% is the first preservative free formulation of a PGF2a analog preparation; it was demonstrated to be well tolerated in naive patients [10] and several studies showed an improvement in tear osmolarity, BUT, tear fluid amount and tolerability in patients who switched from preserved drops to PF Tafluprost and a good tolerability profile [11, 12].

Beta-blockers don’t induce any major local side effect except for a reduction in tear production linked to beta-adrenergic receptor blockade in the lacrimal glands [13]. However an increased expression of immunoinflammatory markers was observed in the conjunctival epithelium of glaucoma patients treated with non preserved timolol [14] and many side effects have been shown for preserved beta-blockers, such as a reduction in corneal sensitivity, dry eye, punctate keratitis, conjunctival hyperemia [3, 15, 16], loss of goblet cells in the conjunctiva [17].

Side effects of preservatives on corneal surface and tear function have been widely shown in literature, it’s not the same as regards active compounds themselves. To our knowledge this is the first work which analyzes in vivo and compares the effects of PF formulations of Timolol and Tafluprost 0.0015%.

## Methods

The purpose of this study was to evaluate and compare the long term Ocular Surface Disease (OSD) signs, in order to evaluate the safety, and symptoms, so to assess tolerability, of Tafluprost 0.0015% and of preservative-free timolol maleate 0.1% in OH and POAG patients by means of clinical tests (Schirmer test I, BUT), in vivo confocal microscopy and Ocular Surface Disease Index (OSDI) and Glaucoma Symptoms Scale (GSS) questionnaires. Systemic side effects were also recorded so to assess general safety.

In our study the safety of the medications concerns the presence and amount of corneal and tear function side effects as well as any systemic adverse event. The tolerability of the drops, which represents the degree to which overt adverse effects can be tolerated by the subject, is strictly linked with the adherence to the therapy and was evaluated by the two questionnaires.

The study was carried out in accordance to the tenets of the Declaration of Helsinki and it was approved by the Local Ethical Committee. Informed consent was obtained from all the subjects after the explanation of the nature and possible consequences of the study.

In this single-masked, observational, cross-sectional study three groups of subjects in care at the Glaucoma service, Eye Clinic, University of Torino, were enrolled:

- Group 1: 54 eyes of 27 ﻿patients on topical﻿ PF Tafluprost 0.0015% monotherapy for at least 36 months;
- Group 2: 48 eyes of 24 patients on topical preservative free Timolol maleate 0.1% monotherapy qd for at least 36 months;
- Control Group: 40 eyes of 20 healthy volunteers, age and sex matched who met the following eligibility criteria: negative history for inflammatory eye disease, previous eye surgery (except for uncomplicated cataract surgery within the last 6 months), ocular trauma, allergic mucosal pathology. These subjects didn’t use topical eyedrops (including artificial eye drops) or contact lens and didn’t suffer from current or previous local or systemic disease that could involve the cornea.

The inclusion criteria for Group 1 and 2 were the following: age 18 years or older, diagnosis of POAG or OH on preservative free PGA or timolol eye drops monotherapy for at least 36 months.

The exclusion criteria for Group 1 and 2 were the following: previous history of intraocular surgery (except for uncomplicated cataract surgery within the last 6 months) or argon laser trabeculoplasty, contact lenses use, recent ocular inflammation, previous or current use of other eyedrops including artificial tear therapy, systemic therapy known to alter tear production, autoimmune diseases, previous severe ocular trauma, any history or biomicroscopy evidence of eye surface impairment.

All subjects were queried for ocular symptoms by OSDI and GSS questionnaires then they underwent a complete ophthalmic examination, BUT, Schirmer I test, in vivo corneal confocal examination (IVCM) and Goldmann applanation tonometry performed 30 min after previous tests. IVCM was performed with a corneal confocal scanning-laser microscope (HRT II Rostock Cornea Module; Heidelberg Engineering GmbH, Heidelberg, Germany), that uses a 670 nm red wavelenght Helium Neon diode laser source. It is a Class I laser so it doesn’t carry risks of ocular injury.The confocal laser scanning device uses a ×60 objective water immersion lens and a working distance of 0 to 3 mm from the applanating cap. The two-dimensional images acquired are defined by 384 × 384 pixels over an area of 400 μm, with lateral digital resolution of 1 μm/pixel and a depth resolution of 2 μm/pixel. The optical section thickness is 4 μm.

The eye being examined was anesthetized with benoxinate hydrochloride 0.4% (oxybuprocaine hydrochloride, ALFA INTES; Industria Terapeutica Splendore S.r.l, Casoria, Italy) eye drops. A drop of Viscotirs gel (0.2% polyacrylic gel, Medivis, Catania, Italy) was used as a coupling material between the polymethilmethacylate contact cap of the objective and the corneal apex. The examiner aligned the objective lens on the corneal apex while the patient was looking at the flashing light. The objective lens was moved towards slowly until it came in contact with the corneal apex. Several sweeps of the entire depth of the cornea were achieved. The examination took 3 to 5 min and was performed on all the subjects enrolled by the same examiner.

Images were analyzed by the same masked investigator using the same light condition. The best images for each patient were chosen and the mean value of 3 images for each parameter studied was considered for statistical analysis. We analyzed the following parameters:

- Basal Epithelial cell density (cell/mm^2^): the manual cell counting procedure of the software was used to perform the cell count in the 400 μm area analyzed; cells only partially contained in this area were not considered in the count.
- Stromal reflectivity (keratocytes activation): just the anterior stromal layer was considered for the analysis and its reflectivity was evaluated by means of a grading scale from 0 to 4 [3], considering the reflectivity of cellular elements with respect to background.
- Number of sub-basal nerves: sum of the nerve branches longer than 50 μm present in a frame of the sub-basal nerve plexus.
- Sub-basal nerve tortuosity: defined by the frequency and the amplitude of the variations in the nerve fiber orientation. It was assessed by a pre-existing scale (0: straight nerve fibers; 4: highly convoluted nerve fibers) [3]
- Sub-basal nerve reflectivity: classified in 4 grades according to a pre-existing scale (1: low reflectivity; 4: very bright reflectivity) [3].
- Endothelial cell density: assessed by the manual cell counting system of the software in the 400 μm area examined.

### Statistical analysis

Normal distribution of the variables was evaluated using the Shapiro-Wilk W test. On the basis of data distribution, the groups were compared with ANOVA or Kruskal-Wallis test for continuous variables; t-test or Mann-Whitney test and Bonferroni’s adjustment of *p*-values were used for post-hoc comparisons.

Demographic and clinical features of subjects were analyzed by descriptive statistics. OSDI and GSS scores, Schirmer I test results, BUT, basal epithelial cell density, stromal reflectivity, number of sub-basal nerves, sub-basal nerve tortuosity and reflectivity and endothelial cell density were considered for statistical analysis.

*P* values <0.05 were considered statistically significant. Statistical analysis was performed using the software STATA® SE 12.1.

## Results

Seventyone Caucasian subjects were enrolled. Demographic features of the subjects are shown in Table 1.

**Table 1:** Demographic features of subjects enrolled in the study

	PF Tafluprost 0.0015% group	PF Timolol 0.1% group	Control group
Patients	27	24	20
Eyes	54	48	40
Age (years)	65.11 ± 10.16	65.08 ± 10.79	64.85 ± 9.76
Gender (male/female)	15/12	13/11	12/8
Diagnosis	54POAG	46POAG/2OH	/
Treatment Duration (months)	42.93 ± 6.04	44.38 ± 8.22	-
IOP (mmHg)	16.61 ± 2.01	16.31 ± 2.86	14.50 ± 1.93

Age, treatment duration and IOP are expressed by mean ± standard deviation

*PF* preservative free, *IOP* intraocular pressure

Mean age and gender were not significantly different between the three groups.

Clinical data (Schirmer I test and BUT), questionnaires scores and confocal microscopy data comparisons between therapy groups, between Tafluprost and control groups and between PF Timolol and control groups are reported in Table 2.

**Table 2:** OSDI and GSS scores, clinical data, in vivo confocal microscopy

	OSDI score	GSS score	Schirmer I test (mm/5min)	BUT (sec)	Basal epithelial cell density (cell/mm^2^)	Stromal reflectiviy (grades 1 to 4)	Number of sub-basal nerves	Sub-basal nerves tortuosity (grades 0 to 4)	Sub-basal nerve reflectivity (grades 0 to 4)	Endothelial cell density (cell/mm^2^)
PF Tafluprost 0.0015% group	10±7.01	85.88±10.92	16.48±8.5	10.09±3.23	5700.91±401.95	2.11±0.82	4.33±1.39	2.17±0.8	2.07±0.8	2476.17±300.83
PF Timolol 0.1% group	12.01±8.74	84.79±11.93	14.13±10.53	10.31±2.86	5745.27±482.24	2.17±0.93	4.23±0.97	2.0±1.01	2.04±0.82	2394.13±310.88
Control group	0±0	99.75±1.10	15.63±4.41	12.12±1.81	5535.43±69.43	1.65±0.70	4.78±0.8	1.3±0.91	1.8±0.85	2360.80±54.65
*P* value										
PF Tafluprost 0.0015% vs PF Timolol 0.1%	0.260	0.666	0.059	0.551	0.535	0.879	0.602	0.377	0.766	0.202
PF Tafluprost 0.0015% vs controls	< 0.0001	< 0.0001	0.548	0.261	0.037	0.006	0.037	<0.0001	0.126	0.105
PF Timolol 0.1% vs controls	<0.0001	<0.0001	0.118	0.026	0.014	0.008	0.003	0.002	0.223	0.715

Differences in OSDI and GSS score, Schirmer I test, BUT and basal epithelial cell density were tested by Mann Whitney test. Differences in stromal reflectivity, number of sub-basal nerves, sub-basal nerve tortuosity, sub-basal nerve reflectivity and endothelial cell density were tested by T-test. *P* values <0.05 were considered statistically significant

*OSDI* Ocular Surface Disease Index, *GSS* Glaucoma Symptoms Scale, *BUT* break up time, *PF* preservative free

Clinical data, OSDI and GSS scores, confocal microscopy parameters (basal epithelial cell density, number of sub-basal nerves, stromal reflectivity, sub-basal nerve tortuosity, sub-basal nerve reflectivity, endothelial density) were not significantly different between the two therapy groups (*p* > 0.05). Conversely the number of sub-basal nerves was significantly higher in controls rather than in Tafluprost or PF Timolol groups (*p* < 0.05 and *p* < 0.01 respectively). Epithelial cell density, stromal reflectivity, sub-basal nerves tortuosity were significantly lower in controls than in Tafluprost (*p* < 0.05, *p* < 0.01 and *p* < 0.0001 respectively) and in PF Timolol (*p* < 0.05,*p* < 0.01and *p* < 0.01 respectively) groups.

As regards clinical data, sub-basal nerve reflectivity and endothelial density no difference was found between Tafluprost and control groups (*p* > 0.05). Schirmer I test value, sub-basal nerve reflectivity and endothelial density were not significantly different between PF Timolol and control groups (*p* > 0.05). On the contrary BUT was significantly higher in controls than in PF Timolol group (*p* < 0.05).

Highly significant differences were observed as regards questionnaires scores between Tafluprost and PF Timolol groups and controls (*p* < 0.0001). No patient had any systemic side effects. Table 3 shows OSDI scores distribution.

**Table 3:** OSDI scores distribution

	PF Tafluprost 0.0015% group	PF Timolol 0.1% group	Total
0 (n°)	13	12	25
(%)	48.15	50	49.02
1 (n°)	12	7	19
(%)	44.44	29.17	37.25
2 (n°)	2	4	6
(%)	7.41	16.67	11.76
3 (n°)	0	1	1
(%)	0.00	4.17	1.96
Total (n°)	27	24	51
(%)	100.00	100.00	100.00

The overall OSDI score defines the ocular surface as: 0- normal (0-12points) or having 1-mild (13–22 points), 2-moderate (23-32points), 3- severe (33–100 points) disease

**Fig. 1:**
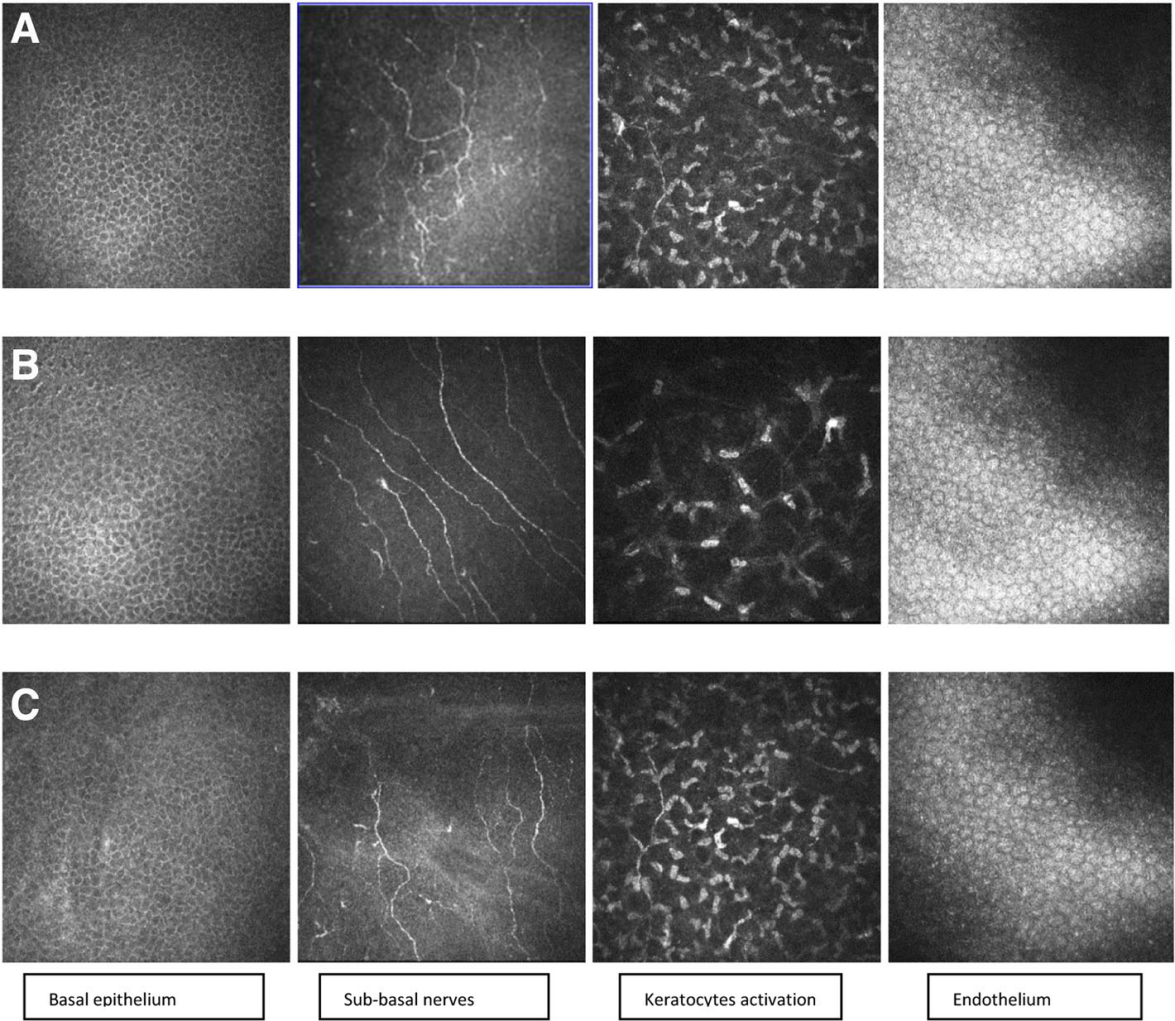
In vivo corneal confocal microscopy findings in Tafluprost 0.0015% group, controls and PF Timolol 0.1% group. **a** Tafluprost 0.0015% group; **b** Control group; **c** PF Timolol 0.1% group. The basal epithelium layer images show a significant increase of cell density in therapy groups with respect to control group; no difference appears between Tafluprost and PF Timolol groups. The sub-basal nerve plexus figures show a reduction of the number of nerve fibers with higher tortuosity scores in patients on therapy with respect to controls; Tafluprost and PF timolol groups don’t differ in sub-basal nerve number and morphology. Stromal images underline a significantly higher keratocytes hyperreflective network in therapy groups than in controls. Stromal reflectivity is similar in Tafluprost and PF Timolol groups. The endothelial images show a similar mosaic pattern in the three groups

**Fig. 2:**
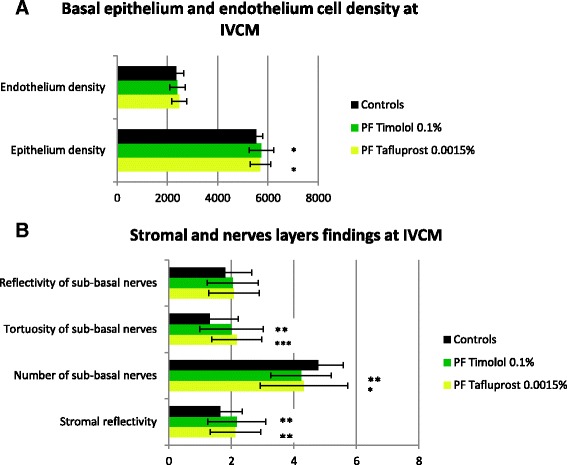
Values of IVCM parameters in PF Tafluprost 0.0015%, PF Timolol 0.1% and control groups. **a** Epithelium and endothelium cells densities. **b** Stromal reflectivity and sub-basal nerves fibers parameters. Statistical significance between each therapy group and controls: *p < 0.05. **p < 0.01. ***p < 0.0001

Figure 1 shows the increased stromal reflectivity, the lower number of sub-basal nerves and their higher tortuosity in Tafluprost and PF Timolol groups with respect to controls. Figure 2 shows the values of IVCM parameters in the three groups.

## Discussion

To the best of our knowledge no previous studies compared Tafluprost and PF Timolol in terms of tear function and corneal layers morphology; just Chabi et al. compared their tolerability and safety resulting in a similar incidence of drug related adverse events, discontinuation attributable to them and tolerability [18].

Our data show that patients treated either with Tafluprost or PF Timolol don’t differ in clinical signs and symptoms.

On the other hand the comparison with controls reveals significant differences in some corneal parameters analyzed by IVCM suggesting a side effect of the active compounds on the corneal layers after a long term therapy.

The IVCM data of our control group are comparable to those available in literature for IVCM of normal patients. We found significant differences between IVCM parameters of Tafluprost and control groups which confirm previous findings on the proinflammatory effect of PGA [19, 20]. In particular we observed an increase of the activation of anterior stromal keratocytes in accordance with Fogagnolo et al.’s findings [20]. PGA treatments seem to increase inflammatory cytokines in the ocular surface which in turn stimulate metalloproteinases (MMPs) [19]; it was reported that metalloproteinases increase the keratocytes density [21] and cause a thinning of central corneal thickness [19] so we suppose that the corneal alterations observed may be the result of the upregulation of MMPs.

In contrast to our findings a recent paper didn’t observe significant changes in corneal parameters in naive patients after 12 months of Tafluprost therapy [22]. The difference in results may be due to the different IVCM employed and treatment duration; little is known on the time of occurrence of corneal changes after starting antiglaucomatous therapies; it may take the corneal alterations more than 12 months to occur.

Corneal alterations were also found in PF Timolol group, these could be due to the subclinical inflammation present in corneal tissues exposed for a long time to beta-blockers. Baudouin et al. noted that PF Timolol induces an overexpression of inflammatory interleukins in conjunctival epithelial cells [14]. Ishibashi et al. reported that PF Timolol disrupts the corneal epithelial barrier function even though to a lesser extent than preserved Timolol [15]. We found less number of sub-basal nerves and an higher sub-basal nerve tortuosity in PF Timolol group than in controls in accordance with Martone et al.’s findings [3]. We also found an higher stromal activation in PF Timolol group than in controls, in contrast with Martone et al.’s data [3]. These discordant findings may be due both to differences in the study populations (higher duration of therapy in our study) and to the poor interobserver reproducibility of IVCM**.**

Before drawing final conclusions from our confocal data it must be underlined that subclinical corneal patterns were found even in untreated OH and POAG patients, although to a less extent with respect to those observed after 12 months of therapy [20]. Therefore our findings could in part have already existed before the beginning of therapies.

As regards clinical data patients in therapy with PF Timolol showed a significant shorter BUT than controls in accordance with Kuppens et al. [13] whereas Martone et al. reported a reduction of both Schirmer I test and BUT values in PF Timolol patients [3]. On the other hand Tafluprost treated patients showed no alterations of lacrimal function in accordance with the current literature [10, 20, 23]; this might be due to a favourable long term influence of Tafluprost on goblet cells [23].

In spite of the signs observed both therapies have a similar overall good tolerability. OSD detected through the questionnaires may be classified as absent or mild in the majority of cases; none of Tafluprost group and just 6.67% of patients in PF Timolol therapy reported a severe ocular surface disease when queried by OSDI questionnaire.

This study has some limitations, which in part reflect the limits of confocal microscopy. We examined just the central cornea, IVCM is a subjective quali-quantitative exam and the meanings of some findings are still unclear: what nerve fibers tortuosity or reflectivity stand for is debated [3, 24]. Another limitation of this study is that it was not prospective. Finally it’s uncertain if ocular surface and corneal alterations observed in patients treated with preservative free eyedrops are due to the active compound itself or rather the eccipients. Therefore further studies are required to investigate this issue.

## Conclusions

We found some corneal alterations in both groups, so the active compound itself may lead to an ocular surface impairment. Tafluprost doesn’t affect tear stability in contrast with PF Timolol; this fact could be of particular benefit in patients with dry eye disease or with an already disrupted ocular surface. Finally we showed that both IOP lowering drops lead to a low to mild severity of ocular discomfort, without significant differences between them.

In conclusion both PF Tafluprost and PF Timolol have a comparable 36 months safety and tolerability. On the basis of our results clinicians should be aware that even PF formulations may induce OSD even though to a less extent than preserved ones. As recommended by EGS guidelines [25] a careful evaluation of individual target therapy with the least amount of medication to achieve the therapeutic response should be a consistent goal.

## Abbreviations

GSS: Glaucoma symptoms scale
IOP: Intraocular pressure
IVCM: In vivo corneal confocal microscopy
MMPs: Metalloproteinases
OH: Ocular hypertensive
OSD: Ocular surface diease
OSD: Ocular surface disease
OSDI: Ocular surface disease index
PF: Preservative free
PGA: Prostaglandins analogs
POAG: Primary open angle glaucoma
